# Risk Assessment and Regulatory Exploration of the Lubrication Coating on Intravascular Medical Devices

**DOI:** 10.3389/fcvm.2022.883406

**Published:** 2022-05-27

**Authors:** Yun Xu, Zhang-Yin Ming

**Affiliations:** ^1^Center for Medical Device Evaluation, National Medical Products Administration, Beijing, China; ^2^Tongji Medical College, Huazhong University of Science and Technology, Wuhan, China; ^3^Hubei Key Laboratory of Drug Target Research and Pharmacodynamic Evaluation, Wuhan, China

**Keywords:** lubrication coating, intravascular devices, iatrogenic polymer embolism, coating separation, interventional catheter, interventional guidewires, supervision situation, polymer particulates

## Abstract

Lubricious polymer coatings are increasingly used on intravascular devices to facilitate application processes. Although increasing reports about the detachment and subsequent embolism of polymer particles, this iatrogenic polymer embolism has not been paid enough clinical attention for many years. This article reviews the hazard of coating separation and the difficulty to find it. Furthermore, this proposes the scientific evaluation concept and regulatory exploration to solve the problems.

Since the mid-1980s, polymers have been widely used as the lubrication coating on intravascular devices in the fields of interventional heart disease, interventional neurology, interventional radiology, and vascular surgery, thus enhancing the lubricity and biocompatibility of the device surface. Lubrication coating includes hydrophilic polymers such as polyvinylpyrrolidone (PVP), polyacrylic acid, and hydrophobic polymers such as polytetrafluoroethylene (PTFE), which can effectively reduce the friction between the surface of the intravascular device and blood vessels or other intravascular instruments, reduce the risk of vascular wall damage, and prevent vasospasm and thrombosis. Therefore, the application of surface lubrication coating expands the scope of interventional instruments for surgical treatment. It reduces the surgical time and cost, enables several innovative intravascular techniques, and becomes an important tool for minimally invasive interventional therapy (1, 2).

It is of great concern, therefore, that the use of polymer coatings has been observed to result in poor adhesion and separation (flaking, sloughing, and peeling), which can potentially cause adverse events and may have a significant impact on patient turnover (3–7).

## Hazard of Coating Shedding and Why It Is Hard to Identify

### Common Mechanism of Coating Shedding

The disadvantage of polymer coating is that the polymer coating separates and falls during intervention. Mechanical abrasion and time-dependent chemical degradation are the main mechanisms causing the detachment of the polymer coating. Mechanical abrasion is a result of device interaction with the vasculature (e.g., atherosclerotic debris) or other devices used to access or treat a lesion. Mechanical abrasion causes incremental strain on chemical bonds within the bulk polymer structure and between the polymer and the device substrate. Upon reaching a bond-energy threshold, the chemical bonds break, resulting in coating separation (scraping or peeling) from the device. In chemical degradation over time, the bonds within the polymer-coated structure and the bond between the polymer and the substrate weaken with long-term contacting with saline or pulsatile blood. Therefore, the polymer-coating composition and coating-binding mechanism, coating thickness, product storage conditions and shelf life, endovascular operation frequency and proficiency, and the anatomical situation of the target blood vessels can all affect the stability of the coating through mechanical abrasion and time-dependent chemical degradation (8, 9).

### Hazard of Coating Separation

Histopathological observations of patients have raised further concerns about detachment of coatings during clinical procedures. A wide range of documented polymer embolism particulate sizes (cross-sectional sizes ranged from 100 μm to 1.9 mm and length ranged from 8 mm to 2.3 cm) and adverse reactions have been reported in the lungs, brain, heart, kidney, liver, and skin/extremities. It was reported that myocardial ischemia and infarction with polymer-coated embolization in the heart, circular enhancement of polymer particles, and surrounding edema were observed in the brain. In some cases with neurodegenerative changes, newborn polymer embolization and occlusion of dermal vessels resulted in skin damage in the lower limbs, manifested as spots and purple patches (10–12).

### The Difficulty to Identify Coating Separation

In 2009, Rupal et al. first reported the fatal complications of iatrogenic hydrophilic polymer embolism (HPE) to the Food and Drug Administration (FDA) and the current awareness of morbidity and mortality caused by iatrogenic HPE. In fact, foreign bodies in the small arteries after use of an infusion microcatheter was reported in 1997. However, after more than 30 years of interventional coating use, the harm of coating HPE is still not being taken seriously enough. The main reason is that polymer-coated embolization is difficult to detect in the clinical setting. As these foreign bodies were not previously encountered, differences in opinion between physicians and discreet disclosure of details of unexpected negative results prevented and delayed reporting of these cases. In the absence of clinical suspicion of iatrogenic coating complications, or targeted histopathological analysis, HPE has failed to gain full clinical attention and awareness over the years (13–15).

Identification of clinical HPE phenomena requires direct biopsies of important tissues, thorough autopsy analysis of the organs and vasculature, or histological testing of the removed thrombotic/emboli. The low autopsy rate in the hospital and the lack of autopsy procedures make the association between polymer embolism and clinical sequelae challenging. Furthermore, while the histological analysis remains the only clearly diagnostic method, the microscopic performance of endovascular polymers and the limitations of tissue sampling led to frequent false-negative interpretations and significant underreporting. Another reason is that with the same device, different surgeons have different results due to operational proficiency, and the risk of HPE associated with the HPE phenomenon may vary further in different patients due to unique anatomical and clinical considerations.

Therefore, in the absence of clinical coating separation complications and the absence of targeted histopathological analysis, HPE has not been paid enough clinical attention for many years.

## The Supervision Situation and Bottleneck

### Current Supervision Situation

Intravascular interventional devices such as vascular catheters or guide wires are managed as the Class III medical device in European Union and China and as the Class II device in the United States, which are regulatory products that do not need clinical trials. Currently, pre-market evaluation requirements for coated intravascular instruments include coating stability/integrity and lubricity studies and evaluating coating stability/integrity on the surface of the instrument. The applicants basically research the coating durability, friction, and stability with their own methods. The administration authorities usually recommend evaluating the substantial equivalence to the approved products.

However, there are no standard methods for detection of particulates, no permissive size threshold, and strict overall permissive limits for producing particulates. The current focus on the particles is mainly about the control of production environment, process, or particles caused by products and their packaging. Presently, the particle control of products generally follows the standard for small specification injection in the national pharmacopeia. That is, particles each test product contained more than 10 μm should not exceed 6,000, and the particle more than 25 μm should not exceed 600.

However, the evaluation method of the particle does not reflect the real situation of particles in the clinical application of coating products. Polymer embolism raises this issue to new concerns. Thus, the analyses of the distal vasculature, organ-related responses, and/or long-term biopolymer effects have not been specifically evaluated, and so far, their effects are unclear.

### The Bottleneck Faced

There are no internationally recognized standards for the interventional device surface lubrication coating and its performance evaluation. The American Association for the Promotion of Medical Devices (AAMI) uses particle testing as an industry standard for assessing the coating integrity of endovascular equipment, but states in Section 6 of the AAMI TIR 42 “because of the absence of comprehensive and definitive clinical data, particle size ranges and particle count limits are not recommended in this TIR. “The International Organization for Standardization 10993 Standard Series (ISO10993), entitled ”Biological evaluation of medical devices,' puts forth guidelines for preclearance evaluation of hydrophilic vascular medical device biocompatibility and safety, incorporating requirements for preclinical testing of polymeric degradation. Part 13 of the Standard provides general requirements for evaluating particulates released from polymeric medical device surfaces, when subjected to simulated clinical environments. Notably, recommended studies are performed by individual manufacturers *via* non-standardized protocols (16, 17).

In 2015, the FDA issued a document entitled “Safe Communications for Intravascular Medical Device lubrication coating separation” which acknowledged these concerns, reminding the potential hazards and risks of polymer lubrication coatings on vascular interventional devices and suggesting safe clinical practice. It also concluded that it will work with stakeholders to develop non-clinical testing methods, establish performance standards, and identify the gap between the current national and international equipment standards on coating integrity performance. Despite this communication and multiple case reports from physicians, pathologists, dermatologists, and other physician specialties involved, polymer-coated embolization remains clinically under-recognized. In the industry communication, the US FDA recognized the important role of hydrophilic coating of vascular device products. Although its operating difficulties and the subsequent risk, the vascular intervention provides doctors with greater operability and reduces vascular friction rupture and vasospasm. The FDA recommends that manufacturers are responsible for developing an appropriate particulate matter assessment procedure and providing an interpretation of the test data (18, 19).

## Scientific Evaluation Concept and Exploration

### International Progress

On 10 October 2019, FDA issued “Coronary, Peripheral, and Neurovascular Guidewires Performance Tests and Recommended Labeling.” It is recommended to evaluate the coating integrity and particulates and to evaluate the lubrication function of the coating. The coating integrity testing should illustrate the data collected from the device in a model representative of bending and test the particles while conducting the coating integrity test to evaluate the source, number, and size of particles that may be lost during simulation. If there are coating defects, scientific and reasonable instructions should be provided on whether these coatings have safety risks. It is recommended that the number of particles should be quantified by size and quantity in each evaluation, and the quantification method (e.g., light shading and light refraction) should be confirmed (20).

On the same day, FDA issued “Intravascular catheters, wires and delivery systems with lubrication coating-Labeling Consideration.” This safety information was released to further clarify that the hydrophilic and/or hydrophobic coating may fall off the surface of the interventional device and may cause serious harm to the patient. Multiple operations that may reduce the coating stability and the cause coating shedding are warned in the instructions and labels (21).

### Scientific Evaluation and Exploration

How to scientifically guide the evaluation and risk control of the lubrication coating of vascular interventional products, the basic link lies in the establishment of industry-approved standard methods and acceptable particle evaluation indicators through the classification evaluation idea and building a closed-loop evaluation evidence chain without affecting the lubrication performance of the coating.

#### Significance of Classification and Evaluation

Both the stability and lubricity evaluation of the coating involve the test cycles/times, that is, the evaluation that the product can maintain the integrity and lubricity of the coating after several test cycles/times. The significance of classification evaluation is to provide a reasonable evaluation platform for different test cycles/times. For example, a guidewire may be prepped, placed in a saline bath, and inserted and withdrawn repeatedly during a procedure (e.g., chronic total occlusions) and may have a higher durability standard. On the other hand, a transcatheter aortic valve repair (TAVR) device may be prepped and inserted once or twice during a procedure and may have a lower durability standard. Accommodations for clinical, anatomical, and procedural variations are required when determining the durability standard for a device category. For products with different applications, it is recommended to develop different simulation test cycles (times) in combination with clinical practice. Therefore, it is necessary to classify the shedding risk of the product lubrication coating according to the anatomical characteristics of the clinical use and application processes.

#### Consideration of the Coating Thickness

At present, an important factor of coating thickness is not considered in coating evaluation. The coating thickness is not the thicker the better; on the contrary, a thinner coating under the conditions of satisfying lubrication can reduce the risk of coating shedding within the blood vessels.

Measure the amount of equipment coating (thickness) and test its durability by determining the maximum possible cycle count. Compare the cycle counts with predetermined durability criteria to assess the number of coatings on the equipment. Cycle counts significantly higher than the device durability criteria may cause the excess coating. In most cases, the manufacturer's coating application process creates a minimum and actual number of coatings applied to the device; however, some changes may occur. In this case, the maximum cycle counts relative to the minimum number of coatings allowed by using the manufacturer's coating application process can also be determined.

#### Evaluation of Particulates

To accurately evaluate the particulates generated during the use of device, the particles should be characterized after simulated use. The number of particulates generated at each evaluation should be quantified and characterized by size and count using a validated method (e.g., light obscuration and light refraction) under continuous flow conditions to simulate blood flow. Coating stability evaluation also involves test cycles/times. For products with different ranges of application, the simulated test cycles/times of tests should represent the most adverse clinical situation. Therefore, it is appropriate to classify the shedding risk of the product lubrication coating according to the anatomical characteristics of the clinical application and application processes.

#### Special Attention

Due to the unique anatomical and clinical considerations, the risk of HPE associated with the coating shedding phenomenon may have different clinical manifestations and outcomes in different patients. Infants and children will be more susceptible to multifocal foreign body deposition as well as inflammatory and developmental sequelae. Cumulative subclinical responses may lead to increased risk of complications throughout their lifetime. A strict treatment for pediatric devices is performed due to possible long-term effects and unique size considerations as well as the tendency for vessel occlusion in distal small vessels and developmental organs. Therefore, there should be stricter restrictions and clear vigilance in the vascular interventional devices used in pediatrics. Furthermore, embolic events should be more likely to develop symptoms in patients with impaired baseline vascular reserve or coma and should attract particular attention.

## The Progress in Lubrication Coating Technology

There is less advancement in the lubrication coating on intravascular medical devices, but some new technology about lubrication coating emerged recently (22). A novel approach to create mucosa-like conformal hydrogel coating was developed. A thin conformal hydrogel layer mimicking the epithelial layer was obtained by first absorbing microparticles, followed by forming covalent interlinks with the polymer *via* interface-initiated hydrogel polymerization. Applications of the mucosa-like conformal hydrogel coating on the endotracheal tube significantly reduced the intubation-related complications, such as invasive stimuli, mucosal lesions, laryngeal edema, inflammation, and postoperative pain. This study offers a promising prototype for surface decoration of biomedical devices.

## Conclusion

There is no doubt that the introduction of lubrication coating has made great progress in the application of vascular interventional devices and has greatly improved the scope of vascular interventional surgery. However, coating is a double-edged sword. How to make full use of its lubrication advantages should cause the common attention and need the joint efforts of producers, regulators, and clinical users.

## Author Contributions

YX: writing the article. Z-YM: critical revision of the article. Both authors contributed to the article and approved the submitted version.

## Conflict of Interest

The authors declare that the research was conducted in the absence of any commercial or financial relationships that could be construed as a potential conflict of interest.

## Publisher's Note

All claims expressed in this article are solely those of the authors and do not necessarily represent those of their affiliated organizations, or those of the publisher, the editors and the reviewers. Any product that may be evaluated in this article, or claim that may be made by its manufacturer, is not guaranteed or endorsed by the publisher.
